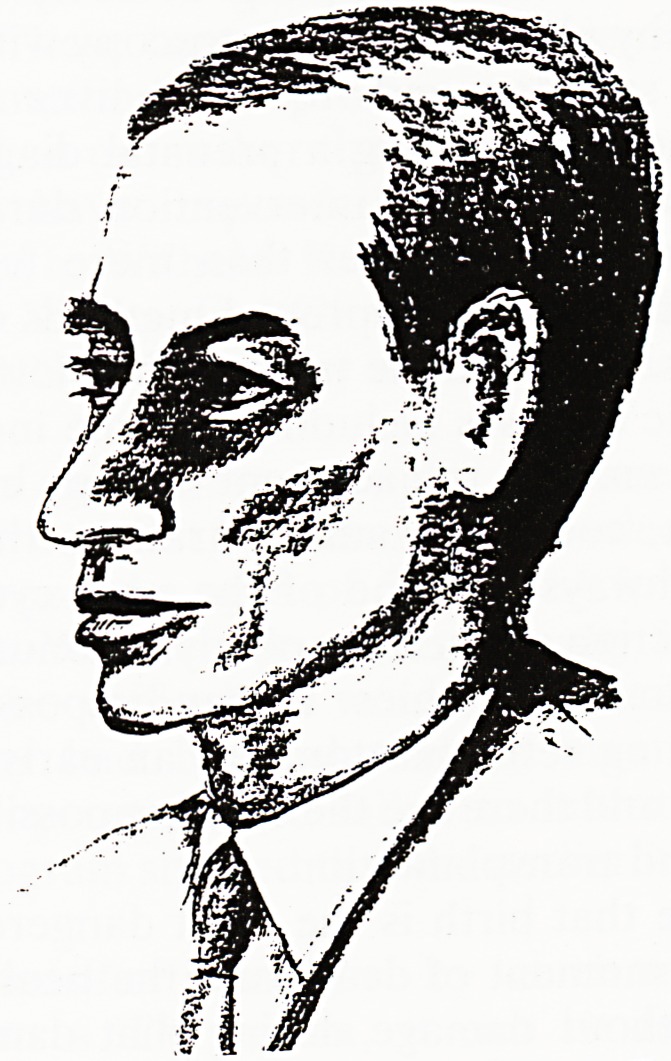# Primary Care in the Future

**Published:** 1988-11

**Authors:** N. C. H. Stott

**Affiliations:** Department of General Practice, Cardiff


### Primary Care in the future

**Professor N. C. H. Stott**
Department of General Practice, Cardiff

Professor Stott began by reminding us that Bristol is the last University in the U.K. to "recognise that academic Primary Care exists" and he said "it does great credit to the Bristol GPs that they had helped to fund the new department". The NHS was built around a generalist service supported by specialists and the U.K. had played a major formative role in the emergence of primary health care worldwide.

The NHS is one of the least expensive health systems in the western world. American experience is very different; by the early 1950s the G.P. was squeezed out by competition between specialists and generalists for clients, with a disastrous escalation of health service costs and major difficulties for the public whose symptoms seldom fall neatly into one specialist category. Inappropriate investigations and procedures became fuelled by a litiginous public, and the USA is now trying desperately to re-establish front line services of a generalist nature. However we in the U.K. should be far from complacent. We are asking ourselves questions about the efficacy of our services. Should nurses be providing more of the service than doctors? Do we need a new kind of primary teamwork for the future?

The public look after 75% of their problems without reference to any doctor. There is a constantly renegotiated interface between the patient and the generalist, and between generalist and specialist, reflecting a willingness to reconsider and renegotiate at different times. Family doctors need to reconsider their role and have the confidence to carry out the treatment of common conditions with full support of the specialist services. No specialist service can cope with chronic conditions occurring in more than 1% of the population except by collaboration with primary health care teams. Advertising by the drug industry is responsible for much inappropriate prescribing and the disappearance of simple and inexpensive remedies. The tranquiliser story speaks clearly of advertising orchestrating specialist opinions to boost sales and general practitioners being left to pick up the mess when specialist views change.

The WHO conference at Alma Ata changed the traditional view of a pyramidal structure for health care and substituted a more horizontal one based on four different approaches — the biomedical approach of appropriate treatment based on accurate diagnosis, the nursing approach which is the provision of continuing care not necessarily concerned with cure, and the social science model which helps us to understand why people seek help, their attitudes to their symptoms, the importance of their fears and their expectations. Finally the health promotion model is widely orchestrated in the White Paper on Primary Care Services and encourages us to take an aggressive approach to people's life style and to screening. If these aspects are thought of as four overlapping circles the heart of the model is where the synthesis of primary careexists with the integration of the four functions which are so important. In isolation biomedicine can lead to inappropriate responses, social sciences can be irrelevant, health promotion a political bandwagon and nursing an indulgence. The heart of family medicine and good primary care is the integration of all four.

Primary health care stands at a new crossroad as performance indicators, strategic goals and measurable objectives have become the language of State sponsored progress. We are being led by the computer industry, by management consultants and by government to eschew our integrated approach and to measure our performance in narrow units of output — immunisation rates, units of minor surgery, numbers of physical examinations, number of prescriptions, referral rates, lifestyles modified and so on. The recent White Paper is quite explicit about these things, the tone was heralded by Maynard from York, one of the leading health economists in our country, who said to general practitioners four years ago that 'clinical freedom is dead, the ethic of the future is efficiency, you are going to suffer questioning and competition for resources like you have never known before, sharpen your knives and get into the fight'. The spectacle of such a fight is deeply troubling, because the primary physician's value system should be one of freedom to respond to needs, and of flexibility to adjust priorities according to competing needs which will always be far greater than the resources to meet them. The caring dimension is easily squeezed out. If narrow performance indicators become the output criteria the vulnerable may suffer most. When clinical freedom dies it usually carries 'caritas' with it.

Performance in primary health care can be viewed from three viewpoints — the consumer's, the professional's and the government's. The consumers take technical competence for granted. What they value is the doctor they can talk to and who will respond quickly and sympathetically to a crisis. Unfortunately most complaints against doctors are the product of failure in these qualities. The qualities are not valued by government as high on their list is business management. Young doctors feel under enormous pressure: the pressure of rewards for activities the public don't necessarily want and the pressure of caring for more and more chronic disease in society. They feel they are being asked to give more of themselves than they are able to — 650,000 people per day see their family doctor in this country. The Government and the public seldom want the same things and there is the fear we could be pushed by market forces to repeat the U.S. disaster when the whole of community care became totally fragmented and the cost of the service soared. If the integrating thinkers have enough influence we can still achieve a balanced service with clinical records reflecting real teamwork, lack of duplication and relevant performance indicators.

A questioner, Professor Drury, the PRCOG asked about the tendency to specialism amongst nurses and ancillary health workers. Professor Stott replied that fragmentation in nursing services could have the same disastrous effect in U.K. as the disappearance of the generalist in the U.S.

## Figures and Tables

**Figure f1:**